# Standardized and accessible multi-omics bioinformatics workflows through the NMDC EDGE resource

**DOI:** 10.1016/j.csbj.2024.09.018

**Published:** 2024-09-27

**Authors:** Julia M. Kelliher, Yan Xu, Mark C. Flynn, Michal Babinski, Shane Canon, Eric Cavanna, Alicia Clum, Yuri E. Corilo, Grant Fujimoto, Cameron Giberson, Leah Y.D. Johnson, Kaitlyn J. Li, Po-E Li, Valerie Li, Chien-Chi Lo, Wendi Lynch, Paul Piehowski, Kaelan Prime, Samuel Purvine, Francisca Rodriguez, Simon Roux, Migun Shakya, Montana Smith, Setareh Sarrafan, Shreyas Cholia, Lee Ann McCue, Chris Mungall, Bin Hu, Emiley A. Eloe-Fadrosh, Patrick S.G. Chain

**Affiliations:** a Bioscience Division, Los Alamos National Laboratory, Los Alamos, NM, USA; b Environmental Genomics & Systems Biology Division, Lawrence Berkeley National Laboratory, Berkeley, CA, USA; c Pacific Northwest National Laboratory, Richland, WA, USA

**Keywords:** Microbiome, Multi-omics, Bioinformatics workflows, Standardization, Software, Open-source

## Abstract

Accessible and easy-to-use standardized bioinformatics workflows are necessary to advance microbiome research from observational studies to large-scale, data-driven approaches. Standardized multi-omics data enables comparative studies, data reuse, and applications of machine learning to model biological processes. To advance broad accessibility of standardized multi-omics bioinformatics workflows, the National Microbiome Data Collaborative (NMDC) has developed the Empowering the Development of Genomics Expertise (NMDC EDGE) resource, a user-friendly, open-source web application (https://nmdc-edge.org). Here, we describe the design and main functionality of the NMDC EDGE resource for processing metagenome, metatranscriptome, natural organic matter, and metaproteome data. The architecture relies on three main layers (web application, orchestration, and execution) to ensure flexibility and expansion to future workflows. The orchestration and execution layers leverage best practices in software containers and accommodate high-performance computing and cloud computing services. Further, we have adopted a robust user research process to collect feedback for continuous improvement of the resource. NMDC EDGE provides an accessible interface for researchers to process multi-omics microbiome data using production-quality workflows to facilitate improved data standardization and interoperability.

## Highlights

• NMDC EDGE is a resource for accessible, standardized microbiome multi-omics workflows.
• Layered software architecture ensures flexibility and enables updates to workflows.
• Feedback is collected through user research efforts to improve the resource.

## 1 Introduction

Multi-omics methods, including a combination of metagenomics, metatranscriptomics, metabolomics, and/or metaproteomics methods, have become more affordable and accessible, enabling new ways to explore diverse microbiomes [25]. Challenges nonetheless exist for researchers to select appropriate computational tools for data analysis and integration, which often require extensive bioinformatics experience and computational resources [20], [51]. Further, the growing number of bioinformatics tools has also resulted in inconsistent data outputs that can be neither compared nor standardized across samples or studies [33]. This limits the generation of Findable, Accessible, Interoperable, and Reusable (FAIR) data, making meta-analyses and machine-learning applications difficult or impossible [55]. Together, these challenges surrounding multi-omics data processing significantly hinder progress in the field of microbiome research.

Web-based resources and cyberinfrastructures are effective ways to promote the accessibility of bioinformatics tools to the larger community because they allow for widespread, on-demand access, reduce the need for specialized local hardware/software installations and maintenance, and foster collaboration by providing centralized platforms for data sharing and tool integration [52], [37], [2], [36]. They can streamline running bioinformatics workflows without the need for local downloads and can be made available to researchers across the globe. However, these systems often require substantial training, an in-depth understanding of computational tools, and the need to weave multiple tools into a workflow to fully leverage these resources.

The Department of Energy’s (DOE) National Microbiome Data Collaborative (NMDC) program strives to provide the microbiome research community with tools and resources that facilitate FAIR data practices [57], [15]. To address shortcomings with many existing bioinformatics workflows and resources, we developed NMDC Empowering the Development of Genomics Expertise (EDGE) to support access to the standardized bioinformatics workflows used to process microbiome data available in the NMDC Data Portal [15]. These standardized workflows developed at two DOE user facilities, the Joint Genome Institute (JGI) and the Environmental Molecular Sciences Laboratory (EMSL), process raw multi-omics data and produce interoperable annotated data from metagenomes, metatranscriptomes, metaproteomes, and natural organic matter characterizations. To date, access to these workflows has largely been limited to the facilities for which they were developed. Herein, we describe the NMDC EDGE resource, which was modeled after the generalized EDGE bioinformatics platform [36] and modified to support a greater volume of projects focusing on microbiome multi-omics data.

## 2 Methods

### 2.1 NMDC EDGE architecture overview

**Fig. 1:**
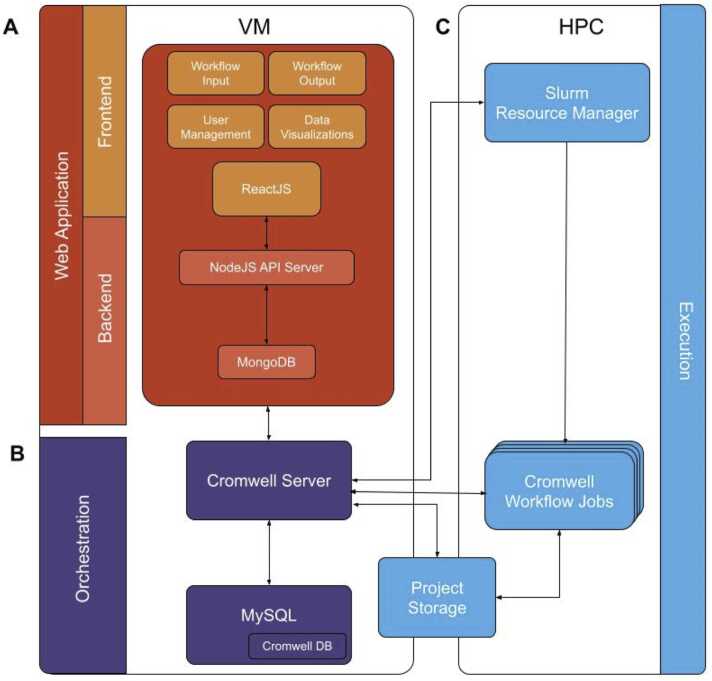
The NMDC EDGE architecture has three layers: the web application (**A**, red), orchestration (**B**, purple), and execution (**C**, blue) layers. The web application layer and orchestration layer run in a virtual machine (VM) and the execution layer runs in a high-performance computing (HPC) environment. (**A**) Users engage with the web application to select input files and workflows. This information is used to populate a workflow template WDL file and an input json file. (**B**) The orchestration layer generates a Cromwell job for the execution layer. Cromwell jobs are executed in a shared Linux computing environment together with non-Cromwell jobs, where the resource manager (e.g., Slurm) handles the actual computing jobs and resources in the cluster. (**C**) Cromwell handles the packaging of WDL-defined workflows into Slurm jobs and also monitors the job execution and updates the MySQL database with the status. When jobs are completed, Slurm resumes control, cleans up temporary files, and writes standard outputs and error messages. Workflow output files are moved to destinations as defined by the WDL files on a filesystem shared between the web application and the execution layer. These then become accessible to the project owner and any users who have been granted access by the owner. The web application layer tracks the status of the workflow (e.g., queuing time, job status) through communications with the orchestration layer and updates the status for the user. In the event of a workflow failure, an error message is displayed to the user, giving information about the source of the failure.

The NMDC EDGE architecture is built using a flexible, modular design, updated from the generalized EDGE bioinformatics platform [36], and is divided into three distinct layers: web application, orchestration, and execution (Fig. 1). This modular design supports updates to workflows and inclusion of new workflows.

### 2.2 NMDC EDGE architecture layers

The **web application** layer (Fig. 1**A**) forms the user interface and is responsible for user interactions. It employs a modern JavaScript web application stack, the MERN (MongoDB, Express.js, React, and Node.js) technical stack, ensuring a user-friendly experience and low maintenance costs [26]. The web application frontend collects user inputs, provides data visualizations and data downloads, and manages user profiles. It is developed with the ReactJS framework with the CoreUI free React admin template (https://coreui.io/product/free-react-admin-template/). The backend of the web application utilizes a MongoDB to store user credentials, input data and workflow outputs, and it controls access to different projects. The frontend communicates with the backend of the web application layer via a set of HTTP Application Programming Interfaces (APIs), ensuring a standard approach to accessing and manipulating resources and a specific backend service that tracks the status and progress of submitted workflows. In terms of extendibility for future additional workflows, this architecture allows for seamless addition of new ReactJS components.

The **orchestration** layer (Fig. 1**B**) manages the bioinformatics workflows and job scheduling. The orchestration layer, supported by the Cromwell workflow manager, manages the execution of complex workflows defined using the Workflow Description Language (WDL)[53]. Cromwell uses a MySQL database for storing execution information, which allows for tracking and management of workflow executions. This setup bridges the gap between the web application layer and the execution layer, providing a workflow orchestration system.

Unlike the web application layer and the orchestration layer that are abstracted from the computing environment, the **execution** layer (Fig. 1**C**), responsible for the actual execution of tasks, interacts directly with the computing resources and job schedulers. NMDC EDGE is compatible and tested in Simple Linux Utility for Resource Management (Slurm) and will also run with other resource management tools supported by Cromwell. By keeping this layer isolated, the architecture remains flexible and is capable of executing tasks without requiring modifications to existing workflows.

To streamline updates and support inclusion of new bioinformatics workflows, all workflow executable files are provided as software containers. Compared to native installations of any bioinformatics workflow, software containers ensure reproducibility and provide increased flexibility for adding new tools and workflows, as they eliminate software incompatibility issues by encapsulating the necessary dependencies and environments. The web application layer and the orchestration layer are deployed to a virtual machine (VM), which has shared project storage space with the high-performance computing (HPC) environment that executes all the workflows.

NMDC EDGE is currently hosted at the San Diego Supercomputer Center (SDSC) and operates within a VM environment with 8 CPUs and 16 GB of RAM dedicated to web hosting and the Cromwell workflow manager. We obtained our allocation on Expanse using ACCESS (Advanced Cyberinfrastructure Coordination Ecosystem: Services & Support) [5]. The workflow manager, in turn, oversees the execution of workflow jobs on the SDSC Expanse cluster computer, which shares a file system with the VM. The workflows are currently executed on Expanse cluster compute nodes with 256 GB of memory. One benefit to the design of NMDC EDGE is its portability across computational resources, where advanced users can download the source code from GitHub (https://github.com/microbiomedata/nmdc-edge). For local HPC installation of NMDC EDGE, users must configure the orchestration and execution layers to work with their existing resource manager and computing environment. For cloud deployments, reconfiguration of the orchestration and execution layers is required, including cloud native code development or replacing Cromwell with the cloud provider's workflow orchestration system, but no changes are needed to the web application layer or the workflow runtime locations.

## 3 Results

### 3.1 The NMDC workflows

**Fig. 2:**
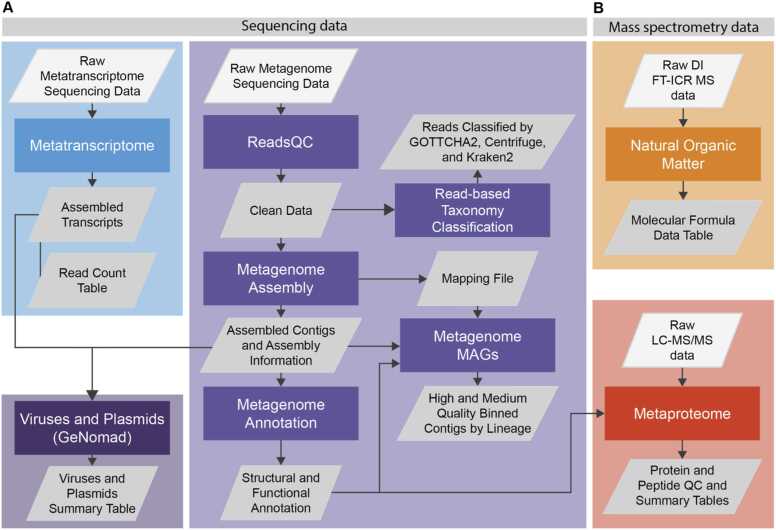
The NMDC standardized bioinformatics workflows and their associated inputs and main outputs for (**A**) sequencing data and (**B**) mass spectrometry data. Additional output files and visualizations are shown in Table 1. The rectangles indicate workflows, and gray parallelograms indicate inputs and outputs. Direct infusion Fourier-transform ion cyclotron mass spectrometry (DI FT-ICR MS); Metagenome-assembled genomes (MAGs); Liquid chromatography-tandem mass spectrometry (LC-MS/MS).

Currently, NMDC EDGE offers five main workflows for processing sequence data (metagenome, metatranscriptome, and prediction of viruses and plasmids) and mass spectrometry data (metaproteome and natural organic matter characterization) (Fig. 2) [13], [14], [15], [8]. The inputs, tools, and outputs of the workflows are outlined in Table 1, and more detailed information regarding the versions, parameters, and other specifics about the workflows can be found on the NMDC documentation site (https://nmdc-documentation.readthedocs.io/en/latest/index.html) and in the NMDC GitHub (https://github.com/microbiomedata/). Every version of the NMDC standardized workflows has fixed tools, parameters, and underlying databases to maintain standardization across runs.

**Table 1:** The NMDC standardized bioinformatics workflows, their inputs, the tools they are using, and the outputs available through NMDC EDGE, in addition to the downloadable output files.

Workflow		Input	Tools	Outputs	Additional NMDC EDGE Output Visualizations
Metagenome Workflow	Reads QC	Raw Illumina sequencing data (.fastq,.fq,.fastq.gz,.fq.gz)	BBTools: rqcfilter2, bbduk, BBMap [3]	Cleaned data as a compressed interleaved FASTQ file (.fq.gz) and QC statistics (.txt)	QC statistics in a summary table
	Read-based Taxonomy Classification	Illumina data (QC’ed); (.fastq,.fq,.fastq.gz,.fq.gz)	GOTTCHA2 [17], Kraken2 [56], Centrifuge [32]	Profiling results for each tool at 3 taxonomic levels (species, genus, family)	Summary tables, interactive Krona plots[46]

	Metagenome Assembly	Illumina data (QC-ed); (.fastq,.fq,.fastq.gz,.fq.gz)	BBtools: bbcms, bbmap; metaSPAdes [3], [42]	Assembled contigs file, scaffolds file, assembly coverage and description files	Table of assembly statistics

Metagenome Annotation	Assembled contig file (.fasta,.fa,.fna,.fasta.gz,.fa.gz,.fna.gz)	tRNAscan-SE [9], Infernal [43], CRT-CLI [4], Prodigal[22], GeneMarkS−2 [38], LAST[18], HMMER[16], [13]	Structural annotations, functional annotations, KEGG summary, Enzyme Commission summary, gene phylogeny summary	Tables of annotation statistics and features

Metagenome Assembled Genomes (MAGs)	Assembled contigs (.fasta,.fa, or .fna), read mapping file from the assembly (.sam.gz or .bam), functional annotation of the assembly (.gff)	SAMtools[35], MetaBat2[27], CheckM[48], GTDB-TK[10], HMMER[16], Prodigal[22], pplacer[39], FastANI[23], FastTree[49], mash[47]	File of High Quality (HQ) and Medium Quality (MQ) bins as well as other lower quality bins	Summary tables of MAG binning information and quality, Each bin’s annotation results.

Metatranscriptome Workflow	Reads QC	Raw Illumina sequencing data (.fastq,.fq,.fastq.gz,.fq.gz)	BBTools: rqcfilter2, BBMap,[3]	Cleaned data as a compressed interleaved FASTQ file (.fq.gz) and QC statistics (.txt)	QC statistics
	Metatranscriptome Assembly	Illumina data (QC-ed); (.fastq,.fq,.fastq.gz,.fq.gz)	rnaSPAdes [7]	Assembled contigs file of transcripts, scaffolds file, assembly coverage, and description files	Tables of assembly statistics

	Metatranscriptome Annotation	Assembled contig file (.fasta,.fa,.fna,.fasta.gz,.fa.gz,.fna.gz)	tRNAscan-SE[9], Infernal[43], CRT-CLI[4], Prodigal[22], GeneMarkS−2[38], LAST[18], HMMER[16][13]	Structural annotations, functional annotations, KEGG summary, Enzyme Commission summary, gene phylogeny summary	Tables of annotation statistics and features

Read Count	Mapped reads (.bam) and annotation file (.gff)	readCov_metaTranscriptome_2k20.pl (dongyingwu/rnaseqct:1.1)	Read counts for transcripts	Table of transcripts and their read counts

Viruses & Plasmids Workflow		Assembly file from a metagenome, metatranscriptome, or genome assembly workflow (.fasta,.fa,.fna)	geNomad[8], CheckV[44]	List of predicted virus sequences and/or regions, along with confidence scores, annotation, completeness, and contamination estimations. List of predicted plasmids with confidence scores and annotations.	Interactive summary table of information about predicted viruses; interactive summary table of information about predicted plasmids; interactive summary table of virus quality

Natural Organic Matter Workflow		Direct Infusion Mass Spectrum Instrument data (Bruker (.d) and Thermo raw), and/or mass list data (.csv, txt)	CoreMS 1.0[14]	Table of all measured *m/z* and all possible molecular formula assignments for each *m/z*.	Mass Spectrum, Mass Error Distribution, van Krevelen diagram, and Carbon # vs. DBE diagram for each heteroatomic class

Metaproteome Workflow		LC-MS/MS Data (.raw); Assembled contig file (.fasta); functional annotation of the assembly (.gff)	MSConvert[21], [30], MSGF+[31], [41], MASIC [24]	Table of identified peptide sequences, protein table with relative abundance measurements, and functional annotations	Summary table of QC metrics

#### 3.1.1 Metagenome workflow

The NMDC standardized metagenome workflow (Fig. 2**A**) leverages JGI’s production pipeline for short-read data and consists of: reads quality control (QC), metagenome assembly, metagenome annotation, and binning of population genomes to generate metagenome-assembled genomes (MAGs) workflows (Table 1**)** [13], [15].

The reads QC workflow utilizes rqcfilter2 to trim and filter low quality data from raw metagenome Illumina reads (FASTQ files). The workflow additionally removes artifacts, linkers, adapters, spike-in reads, and reads mapping to several hosts and common contaminants. The NMDC EDGE interface provides users with a summary table of QC statistics and a variety of metrics, including the number of reads and bases before and after QC filtering.

The read-based taxonomy classification workflow, which is not part of the JGI production pipeline, utilizes three distinct classifiers - GOTTCHA2, Kraken2, and Centrifuge - to profile quality-controlled reads [17], [56], [32]. The use of three distinct tools is meant to accommodate varied project goals and sequencing approaches that cover a spectrum from high sensitivity to high specificity that is dependent on the algorithms and cut-off levels chosen from different tools. The NMDC EDGE interface also provides summary tables and interactive Krona plots as visual outputs for this workflow [46].

The metagenome assembly workflow uses bbcms, metaSPAdes, and BBMap to run error correction, assembly, and assembly validation, respectively [13]. NMDC EDGE provides an output table of assembly statistics. The metagenome annotation workflow takes in assembled metagenomes and generates structural and functional annotations. The metagenome annotation results in NMDC EDGE include tables of statistics for processed sequences, predicted genes, and general quality information from the workflow. The MAGs workflow uses metabat2 to generate metagenome bins and applies the MIMAG standards using annotated tRNAs, rRNAs, and marker genes with checkM to estimate completeness and contamination and subsequent taxonomic lineage assignment [6], [13], [11]. The MAGs result page in NMDC EDGE provides a summary section with information on binned and unbinned contigs, genome completeness, estimated contamination, and the number of genes present on all bins determined to be high quality or medium quality.

Users can run a single workflow within the metagenome pipeline with the appropriate input files, and the entire metagenome workflow is available to run from start to finish on NMDC EDGE from a single input raw Illumina file (Fig. 2**A**). Upon completion of the run, users can view the results, which are grouped by individual workflow.

#### 3.1.2 Metatranscriptome workflow

The NMDC standardized metatranscriptome workflow (Fig. 2**A**) leverages JGI’s production pipeline and consists of: reads QC, metatranscriptome assembly, annotation, and read count workflows. Similar to the metagenome workflow, the reads QC workflow utilizes rqcfilter2 but also removes ribosomal RNA reads. The metatranscriptome assembly workflow uses rnaSPAdes [7] for assembly and uses BBMap to map the reads back to contigs. Assembled transcripts are then annotated with the metagenome annotation workflow described above. Next, reads are counted by mapping to sense and antisense direction of annotated features (e.g., coding sequence or CDS) and unannotated features (e.g., intergenic region). The workflow result page includes a table of the top 100 expressed genes as measured by their read counts. Selecting the header of each column will sort the data by that column. Users can also download a .tsv file of all detected features in the input dataset for further analysis.

#### 3.1.3 Viruses & plasmids workflow

This workflow uses the newly developed geNomad tool [8] to detect putative viruses and plasmids from metagenome and metatranscriptome data (Fig. 2**A**). The workflow provides quality and confidence information from the outputs of CheckV [44]. In NMDC EDGE, the results are displayed as multiple tables. The first output table includes information about predicted viruses in the input data, including sequence length, topology, coordinates, number of genes, genetic code, virus score, false discovery rate (FDR), number of hallmark genes, marker enrichment, and taxonomy. The second table provides the plasmid prediction summary, which includes information on sequence length, topology, number of genes, genetic code, plasmid score, false discovery rate (FDR), number of hallmark genes, marker enrichment, conjugation genes, and any antimicrobial resistance (AMR) genes present.

#### 3.1.4 Natural organic matter workflow

This workflow leverages EMSL’s CoreMS framework and takes in Direct infusion Fourier-transform ion cyclotron mass spectrometry (DI FT-ICR MS) data that undergoes signal processing and molecular formula assignment [14] (Fig. 2**B**). Time domain data is transformed into the frequency domain and finally into mass-to-charge ratio (*m/z*) domain using Fourier Transform and Ledford based equations [54]. Data is then denoised, followed by peak picking and recalibration using an external reference list of known compounds, and searched against a dynamically generated molecular formula library with a defined molecular search space. The downloadable output file from NMDC EDGE consists of a molecular formula table with several columns representing specific measurements and attributes related to the mass spectrometry data, including measured *m/z,* peak height, the molecular formula candidate, the mass accuracy associated with each molecular formula, the heteroatomic class and a composite confidence score that combines the mass accuracy and the spectral similarity of the fine isotopic structure. All molecular formula candidates are shown for each *m/z* measurement that are possible within the molecular search space and the parameters associated with instrument performance. NMDC EDGE uses the default parameters defined on the CoreMS software; modification of these parameters can be achieved using the enviroMS and CoreMS python packages and docker images [14].

#### 3.1.5 Metaproteome workflow

The metaproteome workflow (Fig. 2**B**) is an end-to-end data dependent acquisition (DDA) workflow for protein identification and relative quantification using bottom-up mass spectrometry (MS) data. The workflow takes in raw liquid chromatography-tandem mass spectrometry (LC-MS/MS) data files and an associated metagenome FASTA file to generate: peptide identifications at the user-specified false discovery rate (FDR) using MSGF+ , relative abundance values derived by MASIC from MS1 area under the curve measurements, and protein functional annotations from the provided metagenome [31], [40], [24]. A QC output is displayed in NMDC EDGE that summarizes the proteomic search results and enables users to quickly gauge dataset quality. Result files are provided as downloadable text files. First, the raw output of the pipeline is provided prior to FDR correction, along with the NMDC-produced FASTA file used for the database search. The FDR corrected results are output at the unique peptide sequence level, as well as at the protein level, rolled up using parsimonious inference and summing peptide level abundance measurements [45].

### 3.2 Running the NMDC workflows in NMDC EDGE

**Fig. 3:**
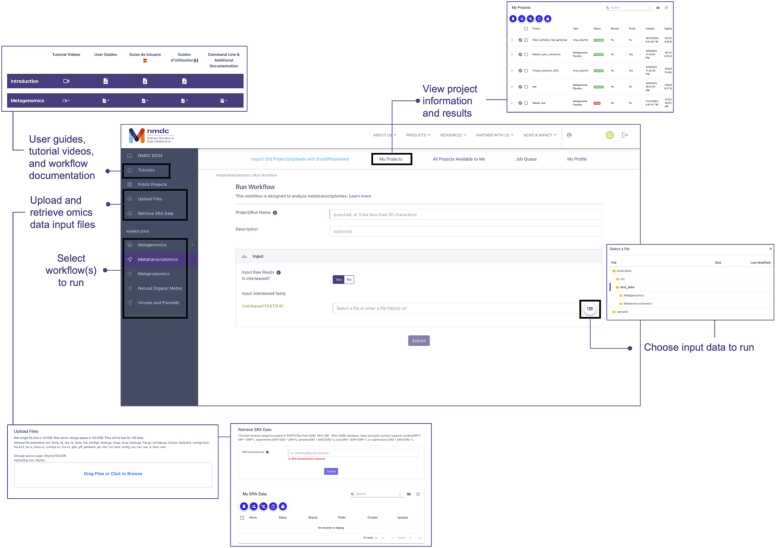
Key features of the NMDC EDGE resource for running standardized NMDC bioinformatics workflows.

The NMDC EDGE resource is open and available at no cost to the microbiome research community at https://nmdc-edge.org (Fig. 3). To run the available NMDC workflows, users must login to the site using ORCiD credentials (https://orcid.org/). Once logged in, the ‘Upload Files’ option allows users to upload omics data files onto the NMDC EDGE resource. The allowable file types are listed on the webpage, and the maximum per-file size is 10.0 gigabytes (Gb) due to limitations of using https to upload data. Raw files will remain on the NMDC EDGE server for 180 days before they are deleted, and users are allocated a total storage space of 150.0 Gb. The limits on individual file size uploads and storage space are comparable to other web-based bioinformatics resources [52], [2]. Users can manage their uploads under the ‘My Uploads’ menu to delete, share, or publish files publicly. Publicly available data files may also be added to NMDC EDGE using the ‘Retrieve SRA Data’ workflow, which imports data housed in the National Center for Biotechnology Information (NCBI) Sequence Read Archive (SRA) directly into NMDC EDGE [34], [28]. Users can then select these datasets as their input data when running a workflow.

Selecting any available workflow will open a webpage where users can input their run information (Fig. 3). Users can select test data, publicly shared data files, SRA data, or privately uploaded files as input to the workflow. Users must also provide any other required information (e.g., if their data is interleaved or the files are paired). Once all required information has been entered and the run has been submitted, the project will appear in the ‘My Projects’ menu (Fig. 3). Within this page, users can view run information, such as the project status (Submitted, In queue, Running, Complete, or Failed) and run type. Users are also given the option to share their project publicly (with all NMDC EDGE users) or to share it with specific NMDC EDGE accounts. Users can select the “View Results” icon to navigate to their results, including summary tables, workflow-specific visualizations, log files, and downloadable output files.

The NMDC EDGE resource uses the FIFO (First-In-First-Out) scheduler to execute jobs in the order they are submitted to the system. The ‘Job Queue’ menu lists all running and pending jobs (Fig. 3). Users can access this menu to see how many projects are running or waiting to run, which can aid in estimating run times. The average runtimes for each of the workflows are listed in Table 2, but the runtimes will vary based on file size, file type, data complexity, and the job queue.

**Table 2:** Average workflow runtimes in NMDC EDGE. For the reads QC, read-based taxonomy classification, metagenome assembly, metagenome annotation, and metagenome MAGs, runtime information from when the workflows were run alone as well as part of the larger metagenome pipeline were combined and averaged to produce the results shown in the table. This information was compiled using the runtime for each task for the 4865 projects that had been run to date when the analysis was performed. File sizes for these projects ranged from 53 B to 13.9 Gb, with an average size of 325 Mb and a standard deviation of 973.6 Mb.

Workflow	Average Runtime (hours)
Metagenome Reads QC	1.82
Metagenome Read-based Taxonomy Classification	0.81
Metagenome Assembly	2.28
Metagenome Annotation	8.85
Metagenome MAGs	0.41
Metatranscriptome	12.64
Viruses and Plasmids	0.97
Natural Organic Matter	0.45
Metaproteome	5.31

### 3.3 Training materials & documentation

A suite of training materials and documentation is available through NMDC EDGE that includes video tutorials, user guides, and technical documentation (https://nmdc-edge.org/tutorial); (https://nmdc-documentation.readthedocs.io/en/latest/tutorials/run_workflows.html) (Fig. 3). Translations of the user guides into Spanish and French are also available. Additional instructional content and descriptions can also be found on the NMDC YouTube channel (www.youtube.com/@microbiomedata) and within publicly available NMDC training materials [29], [50].

### 3.4 User research & usability testing

Since the launch of NMDC EDGE in May 2021, more than 1580 users have collectively run over 6130 workflows. For NMDC EDGE, we employ a user-centered design methodology to collect feedback from the research community, leading to iterative and ongoing improvement to ensure we are meeting the needs of the microbiome research community. Rolling feedback can be submitted via our support email (support@microbiomedata.org) or the feedback form provided on the NMDC EDGE homepage. Beta-testing is an important step of workflow release to the NMDC EDGE interface. Researchers who have contributed NMDC EDGE feedback or participated in beta-testing are acknowledged within this publication. The initial round of beta testing, conducted in 2021, resulted in 49 action items; of these 49, only two were determined to be infeasible by the team. The remaining feedback has been implemented (42 of 49; 86 %) or is in the process of implementation (5 of 49; 10 %). For example, user feedback drove updates to the workflow input pages to clarify the types of acceptable input data, led to the inclusion of clearer avenues for users to reach out to the team for support, allowed the team to identify and fix memory and workflow issues, and led to the improvement of the tutorials and user guides. The most recent round of beta-testing conducted in 2023 (form provided in Supplementary Table 1) resulted in 63 insights and 40 action items. This feedback is actively being discussed, addressed, and implemented.

## 4 Discussion

The NMDC EDGE resource provides bioinformatics workflows using a flexible, modular design for the microbiome research community. These workflows are production-quality, referring to their use by DOE user facility production pipelines which routinely process thousands of datasets. The user-friendly interface has allowed for broader adoption by researchers from various backgrounds to process diverse microbiome datasets. NMDC EDGE supports access to the standardized bioinformatics workflows used to process microbiome data available in the NMDC Data Portal [15]. With the standardized workflows and workflow parameters, researchers can more readily compare their data processed through NMDC EDGE with other datasets available via the NMDC Data Portal, making these datasets more FAIR particularly in regards to their interoperability and reusability.

The standardization and restrictions on customization of workflow parameters allow for more accurate and meaningful comparisons of datasets, however there are inherent limitations with these features. The standardized parameters of the NMDC workflows may not be optimal for all samples, sample types or research questions. Users can retrieve the publicly available NMDC workflows and customize their local runs, however NMDC EDGE has locked down the workflows to allow for seamless comparisons with the projects available on the NMDC Data Portal. Another limitation of the NMDC EDGE resource is that only a limited number of initial workflows are available which may not satisfy all use cases for the broader microbiome research community performing multi-omics and integrative analyses.

New features and improvements are planned for future NMDC EDGE releases. Updates to the existing workflows and their underlying tools and databases will follow a coordinated release schedule with the JGI and EMSL user facility workflows. All workflow and software updates are versioned and tracked in both the software release notes (https://github.com/microbiomedata/nmdc-edge/releases) and the NMDC workflow containers. Currently, NMDC workflows are tested manually. We will implement automated testing and adopt best practices from the software industry, such as continuous integration and continuous delivery/deployment. A new workflow for long-read sequencing data will be added to reflect the growing utilization of this technology for microbiome sequencing [19], [12], [1]. An option for reference-free metaproteome analysis will be incorporated for users to assess the protein composition in their samples without the need for matched metagenomes. A gas chromatography mass spectrometry metabolomics workflow, based on the one in use at EMSL, will be added to NMDC EDGE. Annual rounds of beta testing, user research, and usability testing for the resource will be conducted to continuously improve the workflows, user interface, outputs, and overall user experience. We will work with researchers to successfully apply these workflows to their research, and we aim to make this resource as accessible as possible, regardless of researcher background, location, expertise, or computational resource availability.

## 5 Conclusions

Despite the rapid growth in microbiome research, many barriers exist for multi-omics data processing and standardization. The NMDC EDGE resource provides production-quality bioinformatics workflows in an intuitive web interface with flexibility to support updates and new workflows to be added in the future. Overall, NMDC EDGE serves as a valuable resource to the microbiome research community to facilitate improved data standardization and interoperability.

## CRediT authorship contribution statement

**Yuri E. Corilo:** Writing – review & editing, Supervision, Software, Methodology. **Grant Fujimoto:** Software, Resources, Methodology. **Bin Hu:** Writing – review & editing, Writing – original draft, Supervision, Software, Resources, Methodology, Conceptualization. **Eric Cavanna:** Writing – review & editing, Software, Resources, Methodology. **Emiley A. Eloe-Fadrosh:** Writing – review & editing, Writing – original draft, Supervision, Project administration, Funding acquisition, Conceptualization. **Alicia Clum:** Writing – review & editing, Writing – original draft, Validation, Supervision, Software, Resources, Methodology. **Lee Ann McCue:** Writing – review & editing, Writing – original draft, Supervision, Project administration. **Michal Babinski:** Writing – original draft, Validation, Software, Methodology. **Christopher Mungall:** Writing – review & editing, Supervision, Project administration. **Shane Canon:** Software, Resources, Methodology. **Setareh Sarrafan:** Writing – review & editing, Supervision, Project administration. **Yan Xu:** Writing – review & editing, Writing – original draft, Visualization, Validation, Software, Resources, Methodology, Conceptualization. **Shreyas Cholia:** Writing – review & editing, Supervision, Software, Resources, Project administration, Methodology. **Mark C. Flynn:** Writing – review & editing, Writing – original draft, Visualization, Validation, Software, Resources, Methodology, Funding acquisition, Conceptualization. **Migun Shakya:** Writing – review & editing, Writing – original draft, Validation, Software, Resources, Methodology. **Montana Smith:** Writing – review & editing, Validation, Supervision. **Julia M. Kelliher:** Writing – review & editing, Writing – original draft, Visualization, Supervision, Software, Resources, Project administration, Conceptualization. **Francisca Rodriguez:** Writing – review & editing, Writing – original draft, Visualization, Resources. **Simon Roux:** Writing – review & editing, Writing – original draft, Validation, Software, Resources, Methodology. **Samuel Purvine:** Writing – review & editing, Supervision, Resources, Methodology. **Paul Piehowski:** Writing – review & editing, Writing – original draft, Software, Resources, Methodology. **Kaelan Prime:** Writing – review & editing. **Chien-Chi Lo:** Writing – review & editing, Writing – original draft, Validation, Software, Resources, Methodology. **Wendi Lynch:** Writing – review & editing, Project administration. **Po-E Li:** Writing – review & editing, Writing – original draft, Software, Resources, Methodology. **Valerie Li:** Writing – review & editing, Software, Resources, Methodology. **Leah Y.D. Johnson:** Writing – review & editing, Writing – original draft, Visualization, Software, Resources, Investigation. **Kaitlyn J. Li:** Writing – review & editing, Writing – original draft, Visualization, Validation, Software, Resources, Methodology. **Cameron Giberson:** Software, Resources, Methodology. **Patrick S.G. Chain:** Writing – review & editing, Writing – original draft, Supervision, Software, Resources, Project administration, Methodology, Conceptualization.

## Declaration of Competing Interest

The authors do not have any conflicts of interest to disclose.
